# The Long‐Term Follow‐Up TIPP Project: LOFT Study Protocol, a 20‐Year Prospective Study of Early Psychosis Patients

**DOI:** 10.1111/eip.70127

**Published:** 2026-01-07

**Authors:** Teya Petrova, Philippe Golay, Paul Klauser, Sandra Vieira, Inès Lepreux, Lora Bici, Boshra Razavi, Raoul Jenni, Nadir Mebdouhi, Martine Cleusix, Caroline Conchon, Lilith Abrahamyan Empson, Philippe Conus, Luis Alameda

**Affiliations:** ^1^ Treatment and Early Intervention in Psychosis Program, Service of General Psychiatry Lausanne University Hospital and University of Lausanne Lausanne Switzerland; ^2^ La Source, School of Nursing Sciences HES‐SO University of Applied Sciences Western Switzerland; ^3^ Institute of Psychology, Faculty of Social and Political Science University of Lausanne Lausanne Switzerland; ^4^ Department of Psychiatry, Division of Child and Adolescent Psychiatry Lausanne University Hospital and the University of Lausanne Lausanne Switzerland; ^5^ Center for Psychiatric Neuroscience Department of Psychiatry Lausanne University Hospital and the University of Lausanne Lausanne Switzerland; ^6^ Department of Psychosis Studies Institute of Psychiatry, Psychology and Neuroscience, King's College of London London UK; ^7^ Department of Radiology Lausanne University Hospital and University of Lausanne Lausanne Switzerland; ^8^ Center for Research in Neuropsychology and Cognitive Behavioral Intervention, Faculty of Psychology and Educational Sciences University of Coimbra Coimbra Portugal; ^9^ Centro Investigación Biomedica en Red de Salud Mental (CIBERSAM), Instituto de Biomedicina de Sevilla (IBIS), hospital Universitario Virgen del Rocio, Departamento de Psiquiatria Universidad de Sevilla Sevilla Spain

**Keywords:** early intervention (EI), first episode of psychosis (FEP), long‐term follow‐up, outcome measures

## Abstract

**Introduction:**

Early intervention services (EIS) in psychosis are the gold standard to treat patients after a first episode of psychosis (FEP). However, the understanding of the evolution and the long‐term effects of such type of intervention is limited. This study aims to gain insight into the long‐term evolution of physical and mental health, as well as the neurobiological outcomes of the patients treated for a FEP.

**Methods:**

The Long‐term Follow‐up of TIPP (LOFT) is an up to 20‐year study within a cohort of patients who completed a three‐year EI treatment at Treatment and early Intervention in Psychosis Program (TIPP, in Lausanne, Switzerland) and went through a deep phenotyping prospective multimodal assessment. 720 patients will be contacted and asked to participate in LOFT. Once they are assessed they will be allocated to a timepoint at either 5 (+2), 10 (±2), 15 (±2), and 20 (−2) years after TIPP entry. A follow‐up visit will be proposed every 5 years. All participants will be evaluated on psychopathological, functional, and physical health outcomes including metabolic disturbances. A subsample of the patients who previously took part in a biomarker research program (*n* = 168) whilst at TIPP will be invited to undergo additional assessments (cognition, brain imaging, biofluids collection). Both traditional group‐level and machine learning analyses will be conducted.

**Conclusion:**

Ethical approval has been obtained and recruitment started in 2024, and 111 participants have been recruited so far. LOFT will help reshape and redefine current interventions for subgroups of patients at risk of poorer long‐term outcome and to understand the underlying neurobiological mechanisms influencing psychosis evolution.

## 1 Introduction

Up until 35 years ago, the approach to treating schizophrenia was primarily focused on individuals with severe and chronic disabilities. This paradigm shifted with the development of the early intervention (EI) concept (Falloon 1992), which posits that significant resource investment in the early years of the illness can substantially reduce the number of patients developing chronic disabilities and improve outcomes (Wyatt 1991). It is implied that these early years are critical for intervention, and the potential benefits include reduced morbidity, better prognosis, preserved social skills, and decreased need for hospitalisation (Birchwood et al. 1998). Today, the efficacy of EI for first episode of psychosis (FEP) compared with usual treatment has been proven (Correll et al. 2018), and there are numerous early intervention services (EIS) around the world that are specialised in the detection of the early stages of psychotic disorders, creating avenues for research (e.g., The OPUS trial in Denmark (Petersen et al. 2005), EPPIC in Australia (McGorry et al. 1996), LEO in London (Power et al. 2007)).

While several studies have examined the long‐term outcome of people with psychosis following a FEP up to 10 years (Abdin et al. 2017; Chan et al. 2020; Morgan et al. 2022) or longer (Chang et al. 2019; Cuesta et al. 2023; Suen et al. 2023; Velthorst et al. 2017; Peralta et al. 2025), the understanding of the outcomes in those who received an EI treatment remains insufficient. A systematic review (Chan et al. 2019) revealed that there are 14 published studies that explored the EI outcome at follow‐ups within 10 years (Addington and Addington 2008; Austin et al. 2015; Bergh et al. 2016; Bertelsen et al. 2008; Chang et al. 2015; Gafoor et al. 2010; Hastrup et al. 2013; Hegelstad et al. 2012; Larsen et al. 2011; Mihalopoulos et al. 2009; Norman et al. 2018; Secher et al. 2015), two extending to 12 years (Chan et al. 2018; Sigrunarson et al. 2013). Only one recently published study investigated 20‐year outcomes of patients treated at an EI service during the first years of disease (Hansen et al. 2023). The reported durations of the EI treatment in these studies ranged from 18 months to 2 years, except for one study where specialised treatment lasted 3 years (Addington and Addington 2008). These studies provided useful information on psychopathology (Addington and Addington 2008; Austin et al. 2015; Hegelstad et al. 2012; Larsen et al. 2011; Norman et al. 2018), cost‐effectiveness (Hastrup et al. 2013; Mihalopoulos et al. 2009), optimal EI duration (Chang et al. 2015), cognitive functioning (Bergh et al. 2016), suicide rate (Chan et al. 2018), or a comparison between EI and standard treatment (Bertelsen et al. 2008; Gafoor et al. 2010; Hansen et al. 2023; Secher et al. 2015; Sigrunarson et al. 2013), but none examined neuroimaging, metabolic, and physical health data having shown predictive value for outcome in FEP patients. To date, only one cohort has been comprehensively studied in relation to metabolic health (Vázquez‐Bourgon et al. 2022), neuroimaging (Rodriguez‐Perez et al. 2021), and cognition (Ayesa‐Arriola et al. 2023), 10 years after EI. Moreover, these studies tend to compare the long‐term follow‐up against one baseline measure, precluding predictions at the individual level, which is needed to inform the treatment of patients at the clinical level but requires numerous time point assessments in the early phase of treatment. Finally, long‐term outcome studies of EI programs fail to provide information regarding treatment proposed after specialised EI treatment, which may limit their long‐term benefit. This is a major limitation of current long‐term studies that needs to be overcome.

### 1.1 Early Treatment and Intervention Program

**FIGURE 1:**
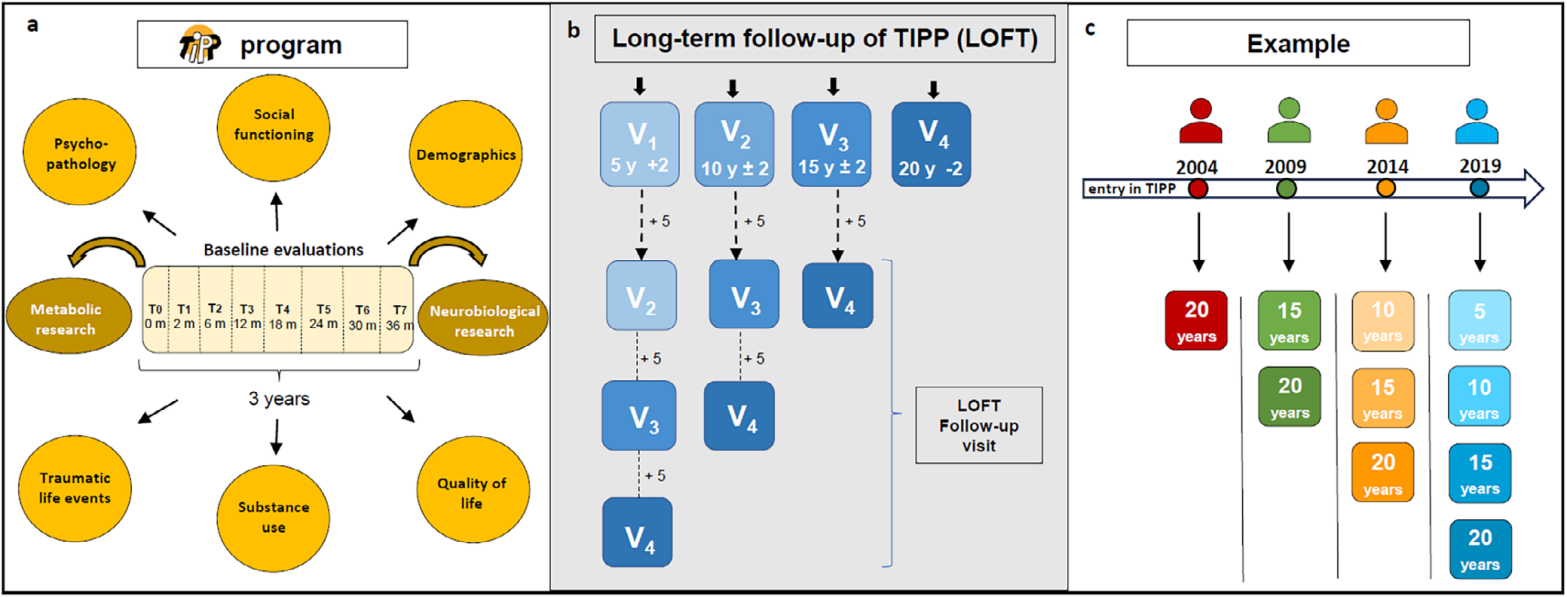
Study design. (a) Baseline evaluations during TIPP medical care. (b) Patients will be contacted and allocated to one of the four different timepoints in regard to the entry date in the TIPP program (T0). Within the timeframe of the study, some of the patients at V1, V2, and V3 will be able to be recontacted for an additional follow‐up visit. (c) Example of entry in the study and timepoint allocation.

The Treatment and Intervention in Psychosis Program (TIPP) in Lausanne, Switzerland (Baumann et al. 2013) was launched in 2004 and is composed of an outpatient case management team and an intensive care mobile team. It is linked to one of the inpatient units focusing on first admissions and patients aged 18 to 35. Covering a catchment area of 330,000 inhabitants, it has treated an average of 46 new FEP patients (aged 18 to 35) each year, resulting in a FEP cohort of close to 1000 patients to this date. The program spans over 36 months and has demonstrated a low disengagement rate (6.3%) (Golay et al. 2020). The patients enrolled in this program are treated within the frame of assertive case management, where specialised nurses work in close collaboration with psychiatrists and psychologists and have an average of 90 contacts with each patient over the treatment period. In addition to treatment, patients go through a detailed multimodal clinical assessment (i.e., psychopathology, social functioning, quality of life, substance use, traumatic life events) at multiple visits (2, 6, 12, 18, 24, 30, and 36 months after entry) throughout the 3‐year treatment duration (Figure 1a), establishing TIPP as likely one of the most robust datasets among various EIS in the world (Baumann et al. 2013).

Recognising the potential metabolic disturbances that can occur as a result of psychopharmacological treatment and poor life style in people with a psychotic disorder (Choong et al. 2008), TIPP patients who were on an antipsychotic medication were also assessed for metabolic parameters to characterise the adverse effects of antipsychotics as part of a local policy to prevent metabolic syndrome (Alameda et al. 2020; Vandenberghe et al. 2015).

Furthermore, a subgroup of patients enrolled in TIPP (*n* = 168) also accepted to participate in a research study targeting a large set of biomarkers. These patients were assessed for a broad range of measures, including neurocognition, brain imaging, electroencephalography, peripheral blood markers involved in, among others, the redox and immune system, as well as for genetics. This subgroup proved to be representative of the whole cohort (Golay et al. 2018).

Therefore, the TIPP data base constitutes a unique three‐year prospective multimodal data set of patients in their first years of treatment after a FEP.

### 1.2 Objectives of the Current Study

The objectives of LOFT are: (i) to assess the long‐term outcomes in TIPP patients and to document treatment received post‐discharge; (ii) to identify both static and dynamic determinants of the long term evolution of psychosis and treatment resistance, encompassing socio‐demographic, environmental and biological factors in order to generate avenues for improvement of early psychosis programs and to gain a deeper insight of the mechanisms of the disease; (iii) to evaluate the long‐term evolution of biomarker profiles (incl. neurocognitive function, brain imaging, electroencephalography and peripheral blood markers) in those patients that were assessed for those variables during their TIPP medical care; (iv) to evaluate physical health outcomes including metabolic syndrome after a first episode of psychosis.

### 1.3 Hypotheses

We hypothesize that (1) early clinical and environmental factors, such as: duration of untreated psychosis (DUP), childhood trauma exposure, and frequent cannabis use, will play a crucial role in shaping the long‐term functional and clinical outcomes, as well as remission rates of patients. (2) The nature of treatment received both during and after early intervention services, including the use of atypical antipsychotic medication and adherence to preventive protocols aiming to minimise metabolic side effects (i.e., metabolic syndrome, obesity‐related problems), is expected to influence patients' clinical trajectories and metabolic health over time. Finally, we anticipate that (3) combining contextual information along with mechanistic dynamic psychological (e.g., mood, anxiety) and neurocognitive (e.g., social cognition) variables that mediate the association between risk factors (e.g., trauma) and poor long‐term outcome will allow the development of accurate long‐term prognostic models. Adding information on biological data involved in the oxidative stress pathway will further improve the models in a subsample of the recruited individuals.

## 2 Methods

### 2.1 Sample

Most patients treated within the TIPP between 2004 and 2019 (*n* = 720) were assessed at baseline with a specially designed questionnaire, which is the TIPP Initial Assessment Tool: TIAT (General Psychiatry Service 2021) which is completed for all patients enrolled in the program by case managers. It evaluates demographic characteristics, psychopathology, past medical history, social functioning, traumatic events history, quality of life, and metabolic parameters. Follow‐up assessments exploring various aspects of treatment and comorbidities as well as the evolution of psychopathology and functional level are conducted by a psychologist and by case managers after 2, 6, 12, 18, 24, 30, and 36 months of treatment (Figure 1a).

#### 2.1.1 Subsample With Neurobiological Data

A subgroup of patients treated in TIPP (*n* = 168) provided additional informed written consent in accordance with our institutional guidelines and local ethics committee to participate in the Biomarker study (Alameda et al. 2018; Aleman‐Gomez et al. 2023; Conus et al. 2018; Dwir et al. 2020; Khadimallah et al. 2022; Klauser et al. 2018; Pistis et al. 2022; Schnider et al. 2022). These patients were assessed at baseline and at 6, 12, and 18 months follow‐up between 2009 and 2021 to investigate a full set of biomarkers, including neurocognition (cognitive and socio‐cognitive abilities), brain magnetic resonance imaging (MRI) (structural, functional, and diffusion), brain MR spectroscopy, electroencephalography (event‐related potentials and time‐frequency activities), peripheral blood markers (transcriptomic, metabolomic, proteomics), as well as genetics (polygenic risk scores). During the LOFT study, they will be invited to undergo the same supplementary exams to constitute a long‐term follow‐up of the neurobiological data collected during their TIPP clinical care.

#### 2.1.2 Subset With Metabolic Data

Patients that were taking antipsychotic medication were evaluated as part of the PsyMetab‐PsyClin cohort (Alameda et al. 2020; Vandenberghe et al. 2015), going through prospective longitudinal monitoring of metabolic parameters including body mass index (BMI), weight gain, and waist circumference. This was conducted at baseline and after 1,2,3,6 and 12 months following the introduction of psychotropic treatment during TIPP. In the LOFT study, all patients will undergo an evaluation for metabolic parameters to obtain a follow‐up measure from the baseline information on physical health.

### 2.2 Inclusion and Exclusion Criteria

TIPP offers 3 years of treatment to patients aged 18–35 years old who meet the following inclusion criteria: (1) meeting the psychosis threshold as defined by the Comprehensive Assessment of At‐Risk Mental States (CAARMS) (Yung et al. 2005); (2) no antipsychotic medication for > 6 months; (3) no psychosis related to intoxication or organic brain disease; and (4) intelligence coefficient ≥ 70. The diagnosis and the date of the psychosis threshold are determined following expert consensus between a senior psychiatrist and a senior psychologist who review the entire files of patients, based on the Diagnostic and Statistical Manual of Mental Disorders, Fourth Edition (DSM‐IV) (Association 2000).

The inclusion criteria for the LOFT study are: (1) having been followed during the TIPP program. Exclusion criteria are (1) inability to provide informed consent, (2) incapacity of discernment, (3) contact is deemed inappropriate based on clinical records or information given by case‐managers who knew the patient, and (4) being deceased.

### 2.3 Recruitment

All patients that have completed the TIPP program assessment will be recontacted by members of the research team. Clinical team members who are familiar with the patient may assist in making this contact. A total of 720 having been part of TIPP between 2004 and 2019 will be contacted between 2024 and 2025 and will be asked to participate in this study. The recruitment started in early 2024. Considering an acceptance rate to enter the research program of ~40% based on previous local studies (Conchon et al. 2023; Wannan et al. 2024), we anticipate that about 288 patients in total will enter the long‐term follow‐up study at four different time points: 5 (+2), 10 (±2), 15 (±2), and 20 (−2) years, depending on the date at which they entered TIPP. Anticipating the same acceptance rate for the subsample, we expect 66 patients from the TIPP‐biomarkers subsample to accept to undergo additional neurobiological evaluations. A follow‐up of the patients that accepted to participate will be proposed every 5 years.

### 2.4 Study Design

This is a longitudinal study, which can be delineated into two phases. The initial phase of recruitment will take place between 2024 and 2025, during which all patients treated in the TIPP program will be contacted by a member of the research team (TP, IL, LB). If they give their consent, they will undergo a series of clinical assessments (Table 1) and will be asked to give a blood sample (Table 2). The patients will be allocated to one of four different time points, either at 5 (+2), 10 (±2), 15 (±2), and 20 (−2) according to when they started the treatment in TIPP (Figure 1b). An example is illustrated in Figure 1c. The TIPP‐biomarker subsample will be invited to undergo additional neurobiological evaluations (Table 3). During the second phase, once all the patients have been contacted, those who have participated in the first visit will be recontacted for an additional visit 5 years later, as detailed in Figure 1b. Analyses mentioned below, however, will be conducted after the first round of assessment.

**TABLE 1:** Clinical assessments.

Domain	Measure	Instrument
Initial evaluation	Demographics	
	Medical & psychiatric history	
	Family psychiatric history	
	Physical health	
	Forensic aspects	
Level of functioning	Psychosocial functioning	GAF (Endicott et al. 1976)
		SOFAS (Morosini et al. 2000)
		GF: Social, GF: Role (Cornblatt et al. 2007)
		MLCI, MVCI (Dion et al. 1988; Tohen et al. 1990)
	Social isolation	The friendship scale (Hawthorne 2006)
	Premorbid functioning	PAS (Cannon‐Spoor et al. 1982)
Psychopathology	Psychotic symptoms	PANSS (Kay et al. 1987)
		BPRS (Overall and Gorham 1988)
	Symptom severity	CGI‐S/CGI‐BP/CGI‐SCH (Busner and Targum 2007)
	Mania symptoms	YMRS (Young et al. 1978)
	Depression	MADRS (Montgomery and Asberg 1979)
	Comorbidities	MINI (Sheehan et al. 1998)
Substance use	Substance use screening	ASSIST (WHO Assist working group 2002)
	Substance use severity	CMRS (Drake et al. 1989)
Social cognition	Theory of mind	RMET (Baron‐Cohen et al. 2001)
Self‐reported questionnaires	Quality of life	WHOQOL‐26 (WHO Assist working group 2002)
	Adverse events	CTQ* (Bernstein et al. 2003)
	Insight	BIS (Birchwood et al. 1994)
	Self‐esteem	RSES (Rosenberg 1965)

*Note:* CTQ will be administered only once during the visit that corresponds to the first follow‐up of the patient.

Abbreviations: ASSIST, The Alcohol, Smoking and Substance Involvement Screening Test; BIS, Birchwood Insight Scale; BPRS, Brief Psychotic Rating Scale; CGI‐S, Clinical Global Impression—Severity; CGI‐SCH, Clinical Global Impression—Bipolar Severity Scale, Clinical Global Impression‐ Schizophrenia severity scale; CMRS, Case Manager Rating Scale; CTQ, Childhood Trauma Questionnaire; GAF, Global Assessment of Functioning; GF: Role, Global Functioning: Role; GF: Social, Global Functioning: Social; MADRS, Montgomery and Åsberg Depression Rating Scale; MINI, Mini International Neuropsychiatric Interview; MLCI, Modified Life Code index; MVCI, Modified Vocational Code Index; PANSS, Positive and Negative Symptom Scale; PAS, Premorbid Adjustment Scale; RMET, Reading the Mind in the Eyes Test; RSES, Rosenberg Self‐Esteem Scale; SOFAS, Social and Occupational functioning assessment scale; WHOQOL, WHO Quality of Life; YMRS, Young Mania Rating Scale.

**TABLE 2:** Biological assessments.

Domain	Measure	
Blood sample	Metabolic parameters	Triglycerides
		Total cholesterol Blood glucose
		HbA1c
		CRP
		Prolactin
Anthropometric measures	Height	
	Weight BMI	
	Waist circumference	
Vital signs	Blood pressure	
	Cardiac frequency	

Abbreviations: BMI, Body Mass Index; CRP, C‐reactive Protein; HbA1C, Haemoglobin A1c; HDL, High‐Density Lipoprotein.

**TABLE 3:** Additional neurobiological assessments.

Type of assessment	Approach/instrument	
Neurocognition	MCBB (Kern et al. 2008; Nuechterlein et al. 2008)	Processing speed
		Attention
		Working memory
		Verbal learning
		Visual learning
		Reasoning and problem‐solving
Brain imaging	Structural MRI Functional MRI Diffusion MRI	Morphometry Functional connectivity Structural connectivity
	Spectroscopic MRI	Metabolic mapping
Electroencephalography	Event‐related potentials	Mismatch negativity
		Sensory gating
	Time frequency activities	Steady states
	Resting states	
Biological assessment	Blood sampling	Transcriptomics
		Metabolomics
		Proteomics
		Genetics

*Note:* Supplementary neurobiological measures collected at baseline from a subsample of the TIPP patients. The patients will be invited to undergo the same exams during their participation in the LOFT project.

Abbreviations: MCBB, Matrics Consensus Cognitive Battery; MRI, Magnetic resonance imaging.

The longitudinal design of this study is a strength, particularly the accumulation of frequent assessments during the first 3 years (at 2, 6, 12, 18, 24, 30, and 36 months of follow‐up). LOFT will then allow additional follow‐up for each individual at 5, 10, 15, and 20 years.

During the first 3 years after a FEP, patients typically undergo intense and dynamic changes in symptoms that shape their clinical trajectory. Because the measurements collected in this early phase are relatively close in time, we will be able to map such changes to early static risk factors (e.g., trauma, cannabis use, duration of untreated psychosis) and to longer‐term outcome measures obtained in the new assessment obtained with LOFT. This longitudinal long‐term design (up to 20 years follow up), with such a rich number of time points is, to the best of our knowledge, unprecedented. It offers an excellent opportunity to test, at the group level (Hypotheses 1–2), how risk factors influence poor outcomes through changes occurring during the first 3 years, and to develop predictive models using both static and dynamic predictors (Hypothesis 3). More concretely for machine learning, this is of particular interest as these models can provide individualised predictions of long‐term outcome that go beyond traditional group‐level associations. Whereas group analyses identify overall patterns and risk factors across the cohort, predictive models allow us to quantify how these factors translate into probabilities for each individual patient. This complementarity is critical in early psychosis, where patients often present with heterogeneous trajectories: group findings inform our understanding of general mechanisms, while predictive modelling translates this knowledge into clinically actionable tools for personalised prognosis and intervention.

### 2.5 Statistical Analysis

#### 2.5.1 Group‐Level Statistical Analyses

Exploratory analyses will be conducted to identify predictors of clinical and functional remission rates. Methods such as Classification and Regression Trees (CART) and random forests will be utilised for comprehensive exploration of the data, without imposing assumptions, ensuring robust and reliable results. Once predictive factors are identified, classical tools such as multiple logistic regression will be employed to estimate predictive power. Due to the longitudinal nature of the data in relation to the baseline TIPP assessments, longitudinal models will be used to describe the clinical and functional evolution in patients and identify factors influencing disease progression. Univariate changes between two measurement occasions will be investigated using repeated measures ANOVA. Multivariate changes will be assessed using latent differences score models. For three or more measurement occasions, multilevel regression models with random slopes and intercepts or mixed effects models for repeated measures analysis of variance will be used to account for both inter‐ and intra‐individual variation over time. Latent growth models will also be used to model individual trajectories.

#### 2.5.2 Predictive Modelling

**FIGURE 2:**
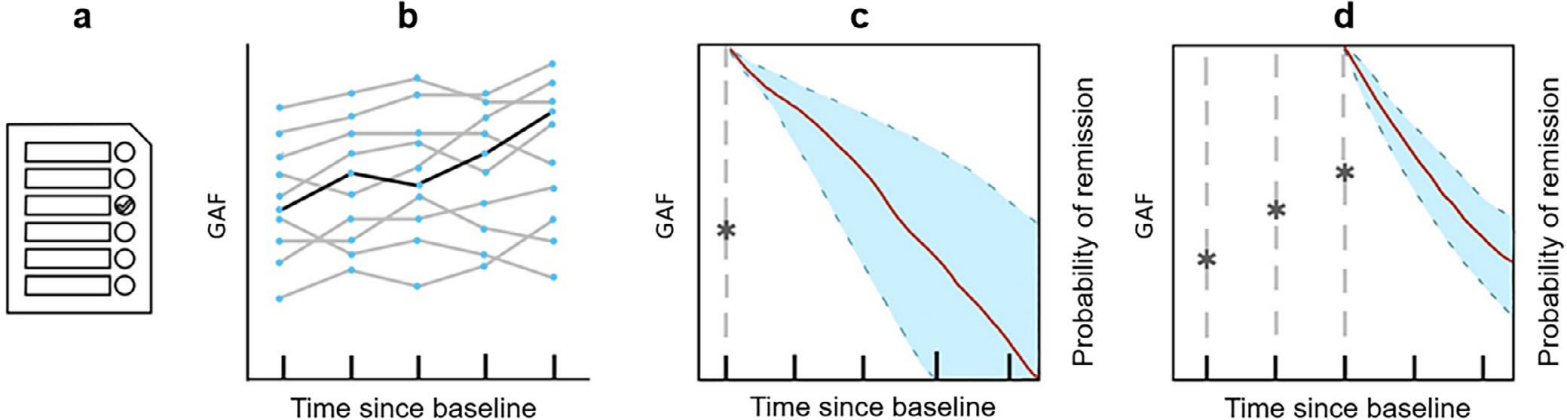
Dynamic predictive model. Example of predictive modelling of GAF scores. (a) Baseline static predictor (childhood trauma collected during treatment in TIPP). (b) Individual trajectories on socio‐occupational functioning (GAF, time dependent predictor) are modelled across all time points via a mixed‐effects model. (c) Individual trajectories are then modelled against remission Cox regression. Predictions using only baseline data (static predictor and GAF) will produce a very high level of uncertainty of predicted probability of remission, as shown by the wide blue margins. (d) As more data is collected across time, the predicted probabilities of remission will become more precise, as shown by the narrower blue margins.

Predictive models will be used to estimate the likelihood of long‐term remission and functional recovery in patients with FEP, with the overarching aim of identifying individuals at greatest risk for poor outcomes and informing personalised intervention strategies (see Figure 2). Firstly, we will use standard supervised methods to investigate the predictive power of early clinical and environmental factors (e.g., DUP, trauma, cannabis use) on long‐term remission and functional outcomes. This will complement group‐level analyses by quantifying how strongly these static risk factors predict individual trajectories and by benchmarking predictive performance using established, interpretable models such as logistic regression, random forests, and gradient boosting. Secondly, we will employ dynamic approaches such as joint modelling to integrate time‐dependent predictors. In this framework, trajectories of repeated measures (e.g., functioning, mood, cognition) will be estimated using linear mixed‐effects models (Fitzmaurice et al. 2012), which are then incorporated into survival models (e.g., Cox regression (Lee and Wang 2003)) to capture their influence on the probability of remission over time. This approach allows predictions to be continuously updated as new data are collected, reflecting the dynamic nature of early illness trajectories. Finally, we will develop multimodal prognostic models by combining contextual, psychological, neurocognitive, and biological data. Early fusion methods, which merge heterogeneous data at the feature level before model training (e.g., canonical correlation analysis (Vieira et al. 2024), principal component analysis), will be used to build joint representations across modalities. In parallel, late fusion methods, which combine predictions from separate models trained on different modalities (e.g., through ensembles or weighted averaging), will be tested to integrate information at the decision level. These complementary strategies will allow us to evaluate whether richer multimodal representations, including oxidative stress biomarkers in a subsample, can further enhance predictive accuracy and clinical utility.

## 3 Discussion and Significance

Ethical approval has been obtained, and recruitment has started in March 2024 (111 patients have been recruited at the time of the submission of the current paper). Funding has been obtained for the first phase of the study (the first 4 years which includes the first 2 years of the first round of recruitment, during which all the patients will be contacted for the first follow‐up visit, which will allow us to address the questions proposed above). Additional funding will be requested in 3 years' time to continue following up with the same participants each 5 years as detailed in Figure 1b, so that each subject has more than one long term follow‐up measurement, providing even richer information on trajectory. To our knowledge, along with OPUS long‐term study (Hansen et al. 2023), LOFT will provide the longest follow‐up of patients treated at an EI service during the early phase of the disease. The data collected from FEP patients at multiple time points during the 3 years of treatment include not only clinical and sociodemographic data but also neurobiological and physical health, making LOFT a unique opportunity to explore outcome in people with psychosis.

We hope LOFT will pave the way for enhancing early psychosis programs and gaining deeper insights into the underlying neurobiological and psychological mechanisms of the progression of the disease, and hopefully to obtain useful predictive models at both individual and group levels.

Conducting such studies is crucial for advancing knowledge and improving outcomes for individuals with psychotic disorders, as well as for subgroups of patients at risk of poorer outcomes including treatment resistance. By identifying modifiable parameters or recognising specific needs, LOFT could enable us to refine clinical practices and move towards precision medicine, tailoring the interventions more precisely to the individual needs of each patient with psychotic disorder from the onset of treatment. In addition to its relevance for individualised treatment approaches, LOFT also holds significance at the group and population levels. By identifying common trajectories, risk and protective factors, this study may inform broader clinical guidelines and thus contribute to the development of standardised care pathways.

Through the utilisation of extensive baseline and follow‐up assessment data and alongside the identification of reliable predictors of long‐term outcomes, this project has the potential of providing valuable predictive algorithms, which may lead to improved treatment and outcomes.

## Funding

This work was supported by Fondation Adrian et Simone Frutiger. Carigest SA. NeuroNA.

## Ethics Statement

The LOFT protocol has been approved on the 17th of October 2016 by the CER‐VD (number of protocol 2016–01354). The use of TIPP clinical data for research purposes and cohort analyses was approved by the local ethical committee Human Research Ethics Committee of the Canton Vaud (protocol #2020–00272). The TIPP and Biomarker studies were approved by the Human Research Ethics Committee of the Canton of Vaud (CER‐VD; protocol #2020–00272 and 382/11).

## Conflicts of Interest

P.C. is an editorial board member of Early Intervention in Psychiatry and a co‐author of this article. To minimise bias, they were excluded from all editorial decision‐making related to the acceptance of this article for publication. The authors declare that they have no other competing interests.

## Data Availability

Data sharing not applicable to this article as no datasets were generated or analysed during the current study.
